# Cardiac Usage of Reducible Poly(oligo-D-arginine) As a Gene Carrier for Vascular Endothelial Growth Factor Expression

**DOI:** 10.1371/journal.pone.0144491

**Published:** 2015-12-09

**Authors:** Jongsu Woo, Seong-Ho Bae, Bokyoung Kim, Jin Sil Park, Subin Jung, Minhyung Lee, Yong-Hee Kim, Donghoon Choi

**Affiliations:** 1 Severance Integrative Research Institute for Cerebral & Cardiovascular Disease, Yonsei University Health System, Seoul, South Korea; 2 Severance Biomedical Science Institute, Yonsei University College of Medicine, Seoul, South Korea; 3 Department of Bioengineering, College of Engineering, Hanyang University, Seoul, South Korea; 4 Cardiovascular Research Institute, Yonsei University College of Medicine, Seoul, South Korea; UCL Institute of Child Health, UNITED KINGDOM

## Abstract

Developments of non-viral carriers have headed toward reducing cytotoxicity, which results from the use of conventional gene carriers, and enhancing gene delivery efficiency. Cys-(d-R9)-Cys repeated reducible poly(oligo-D-arginine) (rPOA) is one of the most efficient non-viral carriers for gene therapy; however, while its efficiency has been verified in the lung and brain, it is necessary to confirm its activity in each organ or tissue since there are differences of gene carrier susceptibility to among tissue types. We therefore tested the compatibility of rPOA in cardiac tissue by *in vitro* or *in vivo* experiments and confirmed its high transfection efficiency and low cytotoxicity. Moreover, substantial regenerative effects were observed following transfection with rPOA/pVEGF expression vector complexes (79% decreased infarct size) compared to polyethyleneimine (PEI) (34% decreased infarct size) in a rat myocardial infarction (MI) model. These findings suggest that rPOA efficiently enables DNA transfection in cardiac tissue and can be used as a useful non-viral therapeutic gene carrier for gene therapy in ischemic heart disease.

## Introduction

Gene therapy is one of the most innovative tools with broad potential application in disease therapy, which might be feasible in the near future. About 450 clinical “gene therapy” trials have been registered in the US National Clinical Trial Database, of which 50 were submitted as studies involving “gene therapy and the heart” [1]. After the first approval of a gene-based therapeutic approach in 2012 [2], several pharmaceutical companies have focused on the clinical application of gene transfer technology, which was used in basic research. Besides canonical gene therapy with viral or non-viral carriers, genome editing has been adopted as gene therapy techniques that attract scientific concern [3]. However, its clinical application remains premature because its safety remains to be verified [4].

Gene therapy vehicles can be classified as viral or non-viral carriers. Several non-viral carriers with numerous new characteristics for gene delivery, including low immunogenicity, safety from random integration into host chromosomes, and easy handling, have been developed [5]. Nevertheless, non-viral carriers are subject to various limitations, such as low transfection efficiency and high toxicity, and temporal gene expression. To overcome these limitations, many researchers focused their efforts on developing optimized enhanced gene carriers [6].

While cardiovascular diseases are promising targets for gene therapy, owing to their high incidence and that only local gene delivery would be necessary to give rise to therapeutic effects [7]. Polyethylenimine (PEI) is a general carrier used in gene transfer to cells or tissues, but is toxic and its use in treatment can often result in cell death [8]. As such, several carriers have been developed to replace PEI, including non-arginine (d-R9) which is known for its efficient protein transduction domains (PTDs) that aid in the delivery of several biological materials (DNA, RNA, proteins, and particles) [9]. Importantly, the Cys-(d-R9)-Cys repeat of reducible poly(oligo- d -arginine) (rPOA) enhances its DNA transfection efficiency[10]. Although the efficacy and toxicity of rPOA has been reported in the lung and brain [10–12], these have not yet been evaluated in the heart.

Myocardial infarction (MI) is a primary cause of heart disease and occurs when blood flow is limited by stenosis or occlusion of the coronary artery restricts the cardiac tissue from receiving sufficient oxygen or nutrients, resulting in cardiac injury [13]. Cardiac tissue injury by irreversible ischemia requires a lifetime of medical treatment. Many researchers have shown that vascular endothelial growth factor (VEGF)-treated infarcted hearts exhibit improved heart function, and thus can be utilized as molecular marker for analyzing gene carrier function [14–16]. Moreover, VEGF secretion is enhanced by the hypoxia-responsive plasmid, pβ-signal peptide-oxygen-dependent degradation-VEGF (pβ-SP-ODD-VEGF), which is induced by post-translational regulation in response to the hypoxic conditions present in MI hearts [17].

In the present study, the feasibility rPOA/plasmid DNA (pDNA) polyplex treatment for MI induced in rat hearts was investigated through a comparative analysis with conventional polyethyleneimine (PEI)/pDNA complexes. These results suggested that rPOA was a suitable carrier for ischemic heart gene therapy.

## Materials and Methods

### Cell culture, *in vitro* transfection, and hypoxia induction

H9c2 rat cardiomyocytes were obtained from the Korean Cell Line Bank (Seoul, Korea) and cultured in high-glucose Dulbecco’s modified Eagle’s medium (DMEM) supplemented with 10% (v/v) fetal bovine serum (FBS) at 37°C in a 5% CO_2_ incubator. For transfection assays, the cells were seeded in wells or culture dishes 24 hours before the transfection. Cells were cultured in serum-free medium for 30 min prior to transfection. For 6-well plates, a fixed amount of plasmid (2 μg/well) was mixed with different ratios (1:1, 1:2, or 1:4) of polymer (2, 4, or 8 μg) in 200 μL of phosphate-buffered saline (PBS), incubated at room temperature for 30 min, and the mixtures were then added to the cells. PEI controls were mixed at a 1:1 ratio. After a 4 h incubation at 37°C, transfected cells were replated in fresh working medium (10% FBS in DMEM). Hypoxia was induced as previously described [18].

### Luciferase assays and flow cytometry

To evaluate gene expression, pCMV-luc plasmid transfected cell lysates were monitored for luciferase activity using a luciferase assay kit (Promega, Madison, WI, USA) according to the manufacturer’s instructions. For flow cytometry experiments, pEGFP-C1-transfected cells were trypsinized, washed with PBS, and the resuspended cells were monitored by flow cytometry (FACSverse, BD science) with the appropriate filters. All experiments were performed in triplicate.

### Cytotoxicity assays

Cell viability was evaluated by 3-[4,5-dimethylthiazol-2-yl]-2,5-diphenyltetrazolium bromide (MTT) assay as described previously [19].

### Rat myocardial infarction model construction and complex injection

*In vivo* experiments were performed with a rat MI model as described previously [19]. The experimental protocol was approved by the Animal Research Committee of the University of Yonsei (Permit Number: 2012–0202). Briefly, eight-week-old male Sprague-Dawley rats were anesthetized by an intramuscular injection of ketamine hydrochloride (90 mg/kg) and xylazine hydrochloride (5 mg/kg). After incision at the left lateral costal rib, the left anterior descending coronary artery (LAD) was ligated with 6–0 silk for 1 h, followed by reperfusion. After surgery, all rats were treated 1 mg/kg ketorolac and 5 mg/kg gentamicin via subcutaneous injection for three days. One hour before reperfusion, 15 μg of plasmid in 50 μL PBS was administered to the myocardium of randomly chosen mice at three sites around the ischemic border and one site within the presumed central infarct region. For the *in vivo* gene expression analysis, animals were re-anesthetized and sacrificed for the fluorescence and histological analysis at three days after surgery. Alternatively, animals were re-anesthetized and killed at one-week after VEGF gene complex infusion to examine VEGF expression and for histological analysis. All histological measurements were performed in blinded manner.

### Fibrosis and infarct size analysis

Hearts were fixed in 10% formalin for 16 h at 2–8°C and then stained with hematoxylin and eosin (H&E) and Masson’s trichrome to analyze tissue fibrosis. Fibrotic planimetry was performed using the ImageJ software (National Institutes of Health, Bethesda, MD, USA). To measure the size of MI, hearts were sectioned transaxially and incubated in 1% 2,3,5-triphenyltetrazolium chloride (Sigma, St. Louis, MO, USA) for 20 min at 37°C. The area of the left ventricle and total collagen-stained area were measured using Image J. Left ventricle infarct size was calculated as the ratio (%) of cumulative infarct area to the entire left ventricle area.

### TUNEL assay

Apoptosis was evaluated using the ApopTag Peroxidase In Situ Apoptosis Detection Kit (Chemicon International, Temecula, CA, USA) according to the manufacturer’s instructions. Briefly, a positive control sample was prepared from a normal heart section treated with 10 U/mL DNase I for 20 min at room temperature. Sections were pretreated with 3.0% H_2_O_2_, reacted with terminal deoxynucleotide transferase enzyme at 37°C for 1 h, followed by incubation with digoxigenin-conjugated nucleotide substrate at 37°C for 30 min. After incubation for 5 min in 3,3-diaminobenzidine (DAB), nuclei exhibiting DNA fragmentation turned dark brown. Tissue sections were counterstained with hematoxylin, mounted, and observed by light microscopy. Five high magnification fields (400×) were examined in each of five sections per group, and the mean of these five measurements was determined (n = 5/group).

### Immunohistochemistry

Immunohistochemistry experiments were performed as previously described [11]. Monoclonal antibodies against CD31 (Abcam, Cambridge, MA, USA) and human VEGF (Abcam) were used at 1:100. The number of CD31-positive cells per mm^2^ was counted in whole sections at 100× magnification in a blinded manner. Images were analyzed using Image J.

### Statistical analysis

Data were analyzed using Student’s *t*-test and presented as the mean ± SD of independent measurements (n > 3 samples/group). P values < 0.05 were considered as statistically significant.

## Results

### Suitability of reducible poly(oligo-d-arginine) for rat cardiomyocyte transfection

To examine the suitability of rPOA for cardiac gene delivery, H9c2 cardiomyocytes were transfected with different rPOA/pDNA ratios (1:0.5, 1:1, 1:2, and 1:4) and analyzed by gel retardation assay. The rPOA conjugates showed complete pDNA condensation at ratios > 1:2 (S1A Fig). The surface properties of these complexes—i.e., particle size, zeta potential, and size distribution—were then investigated. When the weight ratio was > 1:2, the co-polymer completely condensed the DNA and the pDNA size was 170.1 nm (S1B Fig). Moreover, the zeta potentials of the complexes peaked at 43.4 mV resulting from polycation saturation (S1C and S2 Figs).

**Fig 1:**
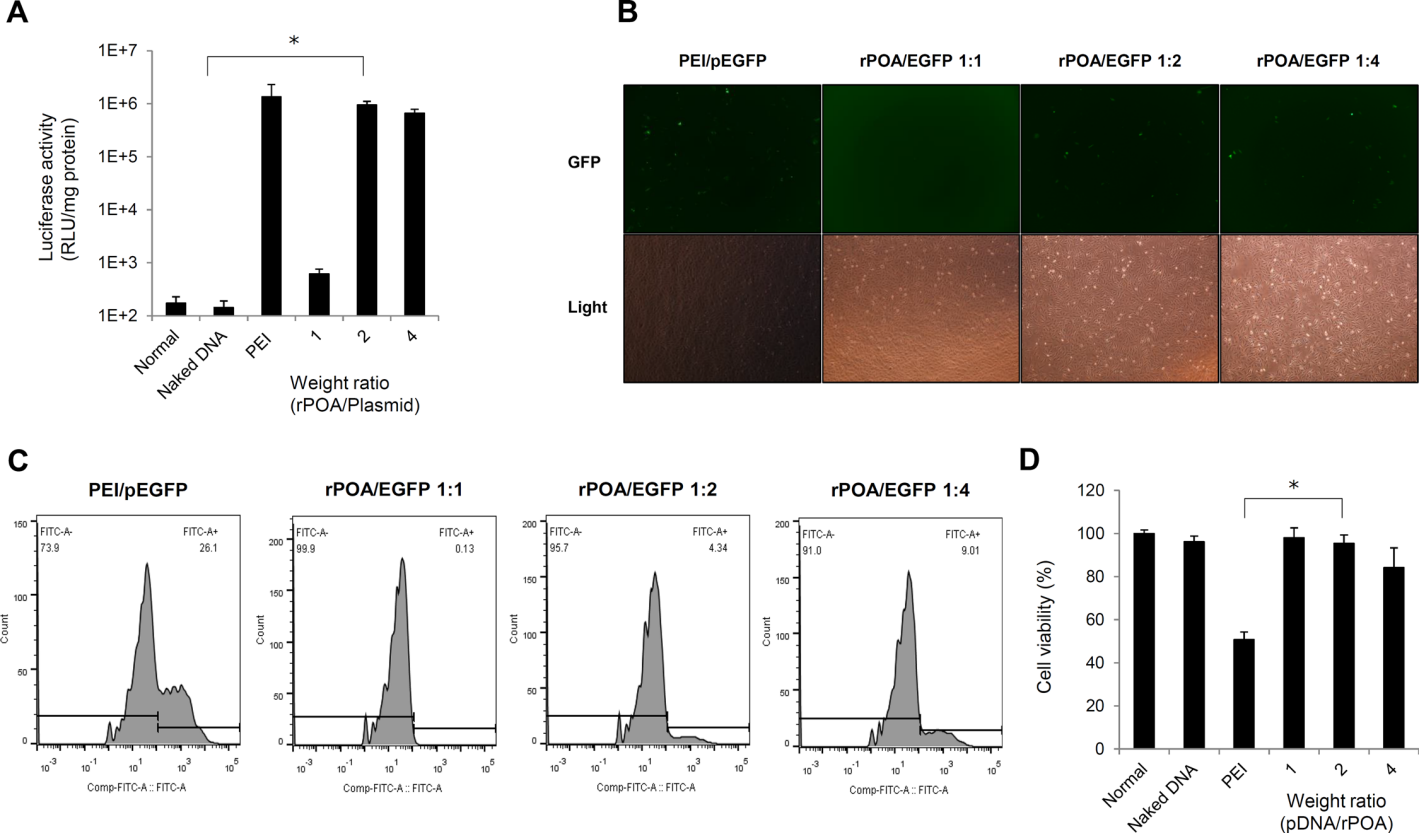
*In vitro* transfection efficiency and cytotoxicity of rPOA as a gene carrier. (A) Optimization of pCMV-Luc transfection into H9c2 cardiomyocytes using three different weight ratios (1:1, 1:2, and 1:4) of rPOA and PEI25K (1:1) as a positive control. Transfection efficiencies were measured by luciferase assay. Data are expressed as the mean ± SD of three experiments. *P < 0.001 vs. PEI. (B) Green fluorescence protein (GFP) expression in rPOA-transfected H9c2 cardiomyocytes. pEGFP-C1 plasmids were mixed with indicated carrier and ratio. A 465–495 nm excitation filter was used to detect GFP expression. (C) GFP-expressing cells were analyzed by flow cytometry to determine the percentage of GFP-expressing cells. (D) Cytotoxicity of rPOA vs. PEI in H9c2 cells. Polymer/pDNA complexes were transfected into H9c2 cells, and cell viability was measured 24 h later by MTT assay. Data are expressed as the mean ± SD. *P < 0.005 vs. control and PEI transfections.

The rPOA/pDNA weight ratio complexes applied to the H9c2 cells and compared to PEI control transfections in luciferase activity assays. Notably, the relative luciferase activity of 1:2 rPOA/pCMV-luc complex-transfected H9c2 cells were similar to that observed in PEI/pCMV-luc complex-transfected cells, but were much higher than the naked DNA- or 1:1 rPOA/pCMV-luc complex-transfected groups (Fig 1A). To confirm the results of the luciferase assay, EGFP-C1-transfected H9c2 cells were analyzed by fluorescence microscopy following transfection with rPOA/pDNA complexes at 1:2 and 1:4 weight ratios. This analysis revealed that a lower number of GFP-expressing cells were detected in the rPOA/pEGFP-C1 complex-transfected group than in PEI/pEGFP-C1 control transfections (Fig 1B). These findings were further confirmed by flow cytometry (Fig 1C), which showed that while ~26% of cells expressed GFP in PEI/pEGFP-C1 transfections, only 9% of cells were positive in the rPOA/pEGFP-C1-transfected cultures.

To determine the cytotoxic effects of these gene carrier complexes, MTT assays were performed with the same experimental groups detailed above (Fig 1D). When 1:2 rPOA/pDNA complexes were applied to H9c2 cells, which showed a transfection efficiency similar to that of the PEI/pDNA complexes, 98% of cells were viable, whereas only 50.78% of PEI/pDNA- transfected cells survived. These data indicate that rPOA shows high transfection efficiency and low toxicity for cardiomyocytes and is suitable carrier for gene delivery in cardiac cells.

### Enhanced *in vivo* gene delivery efficiency with rPOA

**Fig 2:**
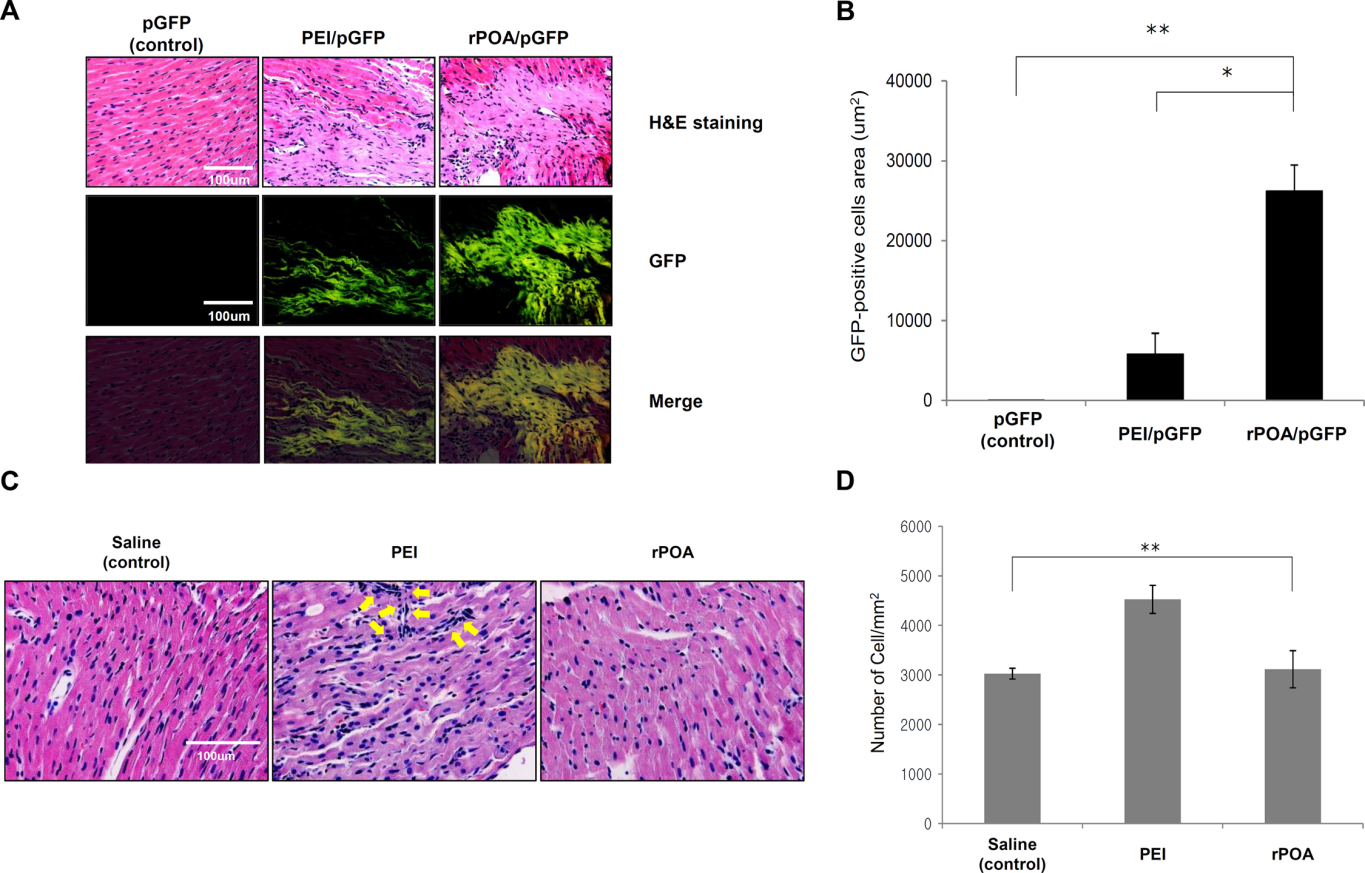
*In vivo* transfection efficiency and cytotoxicity of rPOA as a gene carrier. (A) Representative cardiac tissue sections with H&E staining and GFP fluorescence analysis 3 days after injection of pEGFP-C1, PEI/GFP, or rPOA/GFP into rat hearts (400× magnification). Scale bar, 100 μm. (B) Quantitative analysis of GFP expression across different groups. *P < 0.005, **P < 0.001 vs. control. (C) H&E staining of saline-, PEI-, or rPOA-injected rat myocardial tissue. (D) Quantitative analysis of nuclear stained cells as a measure of left ventricular cellular infiltration post injection with saline (n = 4), PEI (n = 5), or rPOA (n = 6). **P < 0.001 between groups.

To determine the effect of the rPOA/pDNA complexes *in vivo*, rPOA/pEGFP-C1 complexes at the optimized 1:2 ratio were injected into rat hearts to compare the gene delivery efficiency with the PEI/pEGFP-C1 complexes *in vivo*. Three days post-injection, green fluorescence imaging of sliced tissues revealed positive GFP expression (Fig 2). A representative image is shown in Fig 2A, and total GFP expression was quantified and presented in Fig 2B. Notably, the proportion of GFP-positive tissue in rPOA/pEGFP-C1 and PEI/pEGFP-C1 complex-transfected hearts were 26.212 ± 3.271 mm^2^ and 5.773 ± 2.637 mm^2^, respectively.

Cytotoxicity was also analyzed in response to PEI and rPOA treatment in myocardium tissues by monitoring immune cell infiltration with H&E analysis. As expected, the cytotoxic effects of each carrier led to an induction of cell infiltration in target tissues as shown in the arrows indicated area in Fig 2C which shows representative images of immune cell infiltration. In order to analyze these data statistically, all nuclear-stained cells were counted, including all cardiomyocytes. A saline treated group served as normal control (2981.48 ± 150.85 cells/mm^2^). Analysis of the PEI-treated group revealed an increase of ~50% in the cell population (4537.04 ± 283.79 cells/mm^2^). Conversely, a similar but slightly increased cell population was calculated in the rPOA-treated group (3114.82 ± 373.89 cells/mm^2^).

### Application of rPOA in rat model of MI

**Fig 3:**
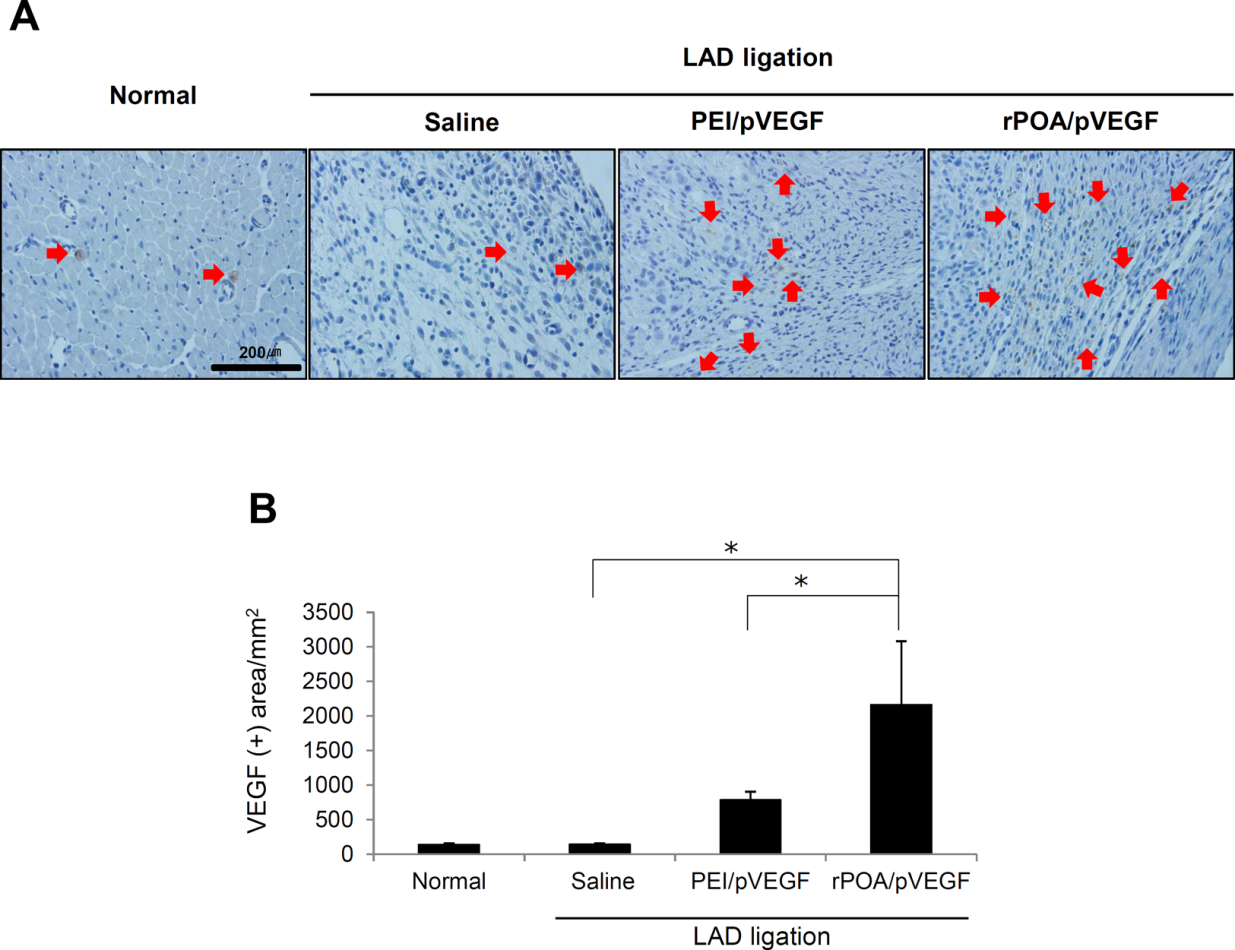
Therapeutic application of rPOA in rat MI model with VEGF expression. (A) VEGF expression in the rat myocardium 1 week after the injection of PEI/pVEGF or rPOA/pVEGF complexes, as visualized by anti-VEGF antibody labeling (200×). Red arrows indicated brown staining of VEGF expression. (B) Quantitative analysis of VEGF immunoreactivity per mm^2^ tissue. Average values were obtained from five random high magnification fields in the infarct border zone from each animal. Data represent mean ± SD. *P < 0.05.

Visualization of VEGF expression by pβ-SP-ODD-VEGF (pVEGF) were verified at the border zone of ischemic cardiac tissue by immunohistochemistry against VEGF proteins (Fig 3). As expected, much higher VEGF expression was detected in the rPOA/pVEGF-injected infarct region than in PEI/pVEGF or control MI group (saline-injected group) (Fig 3A). Quantitative analysis of VEGF immunoreactivity is shown in Fig 3B. Notably, VEGF protein expression was 2.5 fold higher in rPOA/pVEGF-injected tissue than PEI/pVEGF controls (Saline: 135.98 ± 18.58 μm^2^; PEI/pVEGF: 781.41 ± 121.68 μm^2^; and rPOA/pVEGF: 2168 ± 2.7 μm^2^). These data are consistent with the *in vivo* GFP expression observed in Fig 2.

**Fig 4:**
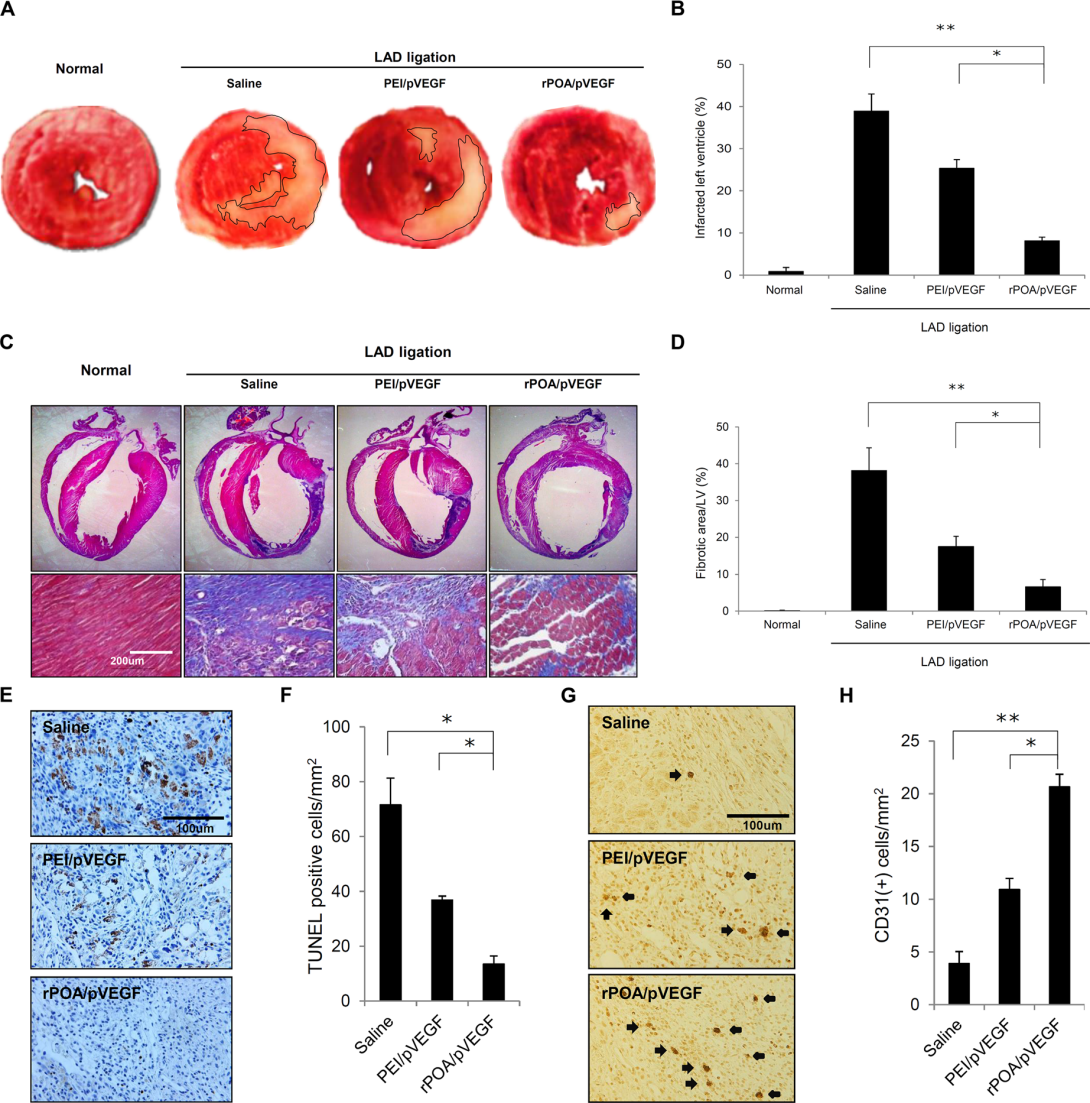
Histological analysis of gene therapy-treated MI hearts. (A) Representative picture of myocardial sections stained with 2,3,5-triphenyltetrazolium chloride (TTC). Pale yellow indicates infarct region. (B) Ratio of infarcted to non-infarcted left ventricular myocardium (%) from LAD-ligated rats injected with saline, PEI/pVEGF, or rPOA/pVEGF (n = 5/group). *P < 0.0005, **P < 0.00001 vs. saline. (C) Representative myocardium sections stained with Masson’s trichrome (lower panel, 200×). Scale bar, 200 μm. (D) Percent of myocardial collagen fibrosis expressed as the ratio of fibrotic area to left ventricle area in LAD-ligated rats injected with saline, PEI/pVEGF, or rPOA/pVEGF (n = 5/group). *P < 0.005, **P < 0.00001. (E) Myocardial sections labeled with an antibody against CD31 to detect neovascularization 1 week after injection (400×). Scale bar, 100 μm. (F) Quantitative analysis of CD31-positive cells in ischemic myocardia treated with saline, PEI/pVEGF, or rPOA/pVEGF (n = 5/group). *P < 0.05, **P < 0.005.

To compare the differences of infarct size among VEGF expression plasmid-injected tissues, the tissues were stained with triphenyltetrazolium chloride (TTC) to highlight the infarcted region with a pale yellow color (Fig 4A). MI-induced hearts were injected with saline, PEI/pVEGF, or rPOA/pVEGF and quantitatively assessed by measuring the area of infarcted tissue area relative to the whole section (Fig 4B). MI-induced cardiac tissue contained approximately 40% of an infarcted region. Notably, the infarct size of PEI/pVEGF-injected tissue decreased to 25.352% ± 2.05% relative to the whole sections. Interestingly, rPOA/pVEGF-injected cardiac tissue revealed an 8.122% ± 0.87% reduction in infarcted tissue. Furthermore, the extent of cardiac fibrosis was analyzed by Masson’s trichrome staining, in which the collagen fibers appear blue (Fig 4C). The percentages of fibrotic area to the left ventricle area were calculated and displayed in Fig 4D. The fibrotic area of saline-injected cardiac tissue was 38.13% ± 6.2%, whereas the PEI/pVEGF-injected cardiac tissue was 17.47% ± 2.78%. Predictably, the fibrosis in rPOA/pVEGF-injected cardiac tissue comprised 6.59% ± 2.02% of the images, which is only 17.29% of that observed in controls.

TUNEL assays were subsequently used to evaluate the extent of cell death at 1 week after LAD ligation. Notably, the number of TUNEL-positive cells in the infarct region was markedly lower upon injection of rPOA/pVEGF as compared to PEI/pVEGF (13.44% ± 3.01% vs. 36.8% ± 1.45% TUNEL-positive cells/mm^2^) (Fig 4E and 4F). To validate direct effects of transferred VEGF gene were measured through angiogenic effects under the direct influence of VEGF. Newly generated capillaries were detected by immunohistochemistry with a CD31 antibody (Fig 4G and 4H). Compared to saline- and PEI/pVEGF-injected groups (3.9 ± 1.13 μm^2^ and 10.92 ± 1.05 μm^2^, respectively), rat hearts injected with rPOA/pVEGF (20.64 ± 1.2 μm^2^) showed a significant increase in micro vascularization within the infarcted myocardial tissue. These data indicate that rPOA/pVEGF transfected cells expressed more VEGF in to the ischemic myocardium than PEI/pVEGF counterparts, resulting in increased capillary generation.

## Discussion

As previously reported, rPOA is one of the most effective non-viral carrier for gene delivery to cultured cells or tissues [10, 12]; however, the efficacy of gene carrier delivery varies according to the cell or tissue type. Gene expression levels in rPOA/pCMV-luc complexes transfected cells measured by luciferase assay showed a similar luciferase activity to that of PEI/pCMV-luc complex-transfected cells, but the analysis of GFP expression by flow cytometry yielded different results since the proteins expressed by transfected gene are the primary therapeutic mediators; thus, a proper evaluation of gene therapy efficiency must be estimated by the amount of protein expressed by the entire cell population, rather than the percentage of cells that express the transfected gene.

The ultimate purpose of gene therapy is clinical application for the treatment of human disease; therefore, the cytotoxicity of the gene carrier is crucial factor to consider. MTT assay showed that rPOA/pDNA-transfected cells exhibited a significantly higher viability (98%) than that observed with PEI/pDNA-transfected counterparts (50.78%). Interestingly, the cells transfected with PEI complexes had to be trypsinized for much longer in order to fully detach from the culture plates, than rPOA-transfected cells. This phenomenon might provide a clue for the increased toxicity of PEI. Although the transfection efficiency of rPOA/pDNA complexes was sufficient to express genes *in vitro*, these findings do not always extend to *in vivo* transfections. The analysis of rPOA/pEGFP-C1 complexes and PEI/pEGFP-C1 complexes *in vivo* revealed a much larger proportion of cardiac tissue with GFP expression in rPOA/pEGFP-C1-injected hearts (26212.95 μm^2^ ± 3271.67 μm^2^) than PEI/pEGFP-C1-injected counterparts (5773.74 ± 2637.52 μm^2^). While the *in vitro* transfection efficiency of rPOA in H9c2 cells is almost same or lower than that observed with PEI, a substantial increase in gene expression was detected in rPOA complex-injected heart tissue. The cardiac tissue contains smooth muscle cells, endothelial cells, epicardial cells, fibroblasts, cardiomyocytes, and pacemaker cells [20], each with their own distinct susceptibility to DNA/carrier complex uptake, which may account for the inconsistencies between our *in vitro* and *in vivo* data.

The pβ-SP-ODD-VEGF plasmid is post-translationally regulated to induce VEGF expression under hypoxic condition [21]. Oxygen deprivation during infarction elicits VEGF protein expression from the pβ-SP-ODD-VEGF plasmid, whereas the protein is rapidly degraded via oxygen-dependent degradation domain during normoxic conditions [22]. *In vitro* and *in vivo* transfections with hypoxic H9c2 cells and ischemic myocardial tissue revealed that this oxygen-responsive system was accurately regulated (S2 Fig). As expected, a markedly higher efficiency of gene delivery was verified with VEGF and GFP detection in rPOA/pVEGF-injected infarcted regions than in PEI/pVEGF or control MI (saline-injected) groups. We analyzed the area of the VEGF positive signal not the number of VEGF expressed cells, because VEGF165, which we used, is delivered to the surroundings from the cells. When the VEGF was expressed in infarcted myocardium, these regions diminished concurrent with improvements in heart function [23]. MI induced cardiac tissue shows about 40% of left ventricle are infarcted (saline injected group). While infarct size in PEI/pVEGF injected tissue (25.352% ± 2.05%) was decreased in about 34% relative to saline-injected tissue, infarct size in rPOA/pVEGF-injected cardiac tissue showed a 79% decrease relative to saline-injected control tissue. Moreover, the extent of cardiac fibrosis revealed a pattern similar to that of TTC staining (Fig 4C), which related to the low number of cells with express transfected gene expression by flow cytometry, but with a high mean value of gene expression in luciferase assays. Gene carrier functions are dependent on two steps; (i) complex formation with nucleic acid, and (ii) penetration of gene into the cells. The number of the transfected cells is dependent on the penetrating ability of the complex in the susceptible point of view, where as its formation determines how many molecules are transferred to a cell in the complex itself available point of view. Therefore, these results suggest that the penetration efficiency is better in PEI complexes, whereas while the complex formation capacity and safety against toxicity is better in rPOA complexes. Thus, the efficient transfer and elevated expression of a gene delivered with rPOA is directly correlated with improved therapeutic effects.

In general, apoptosis is induced in response to MI in human or animal models [24]. The degree of apoptosis can be quantified by counting the number of TUNEL positive cells. As expected, TUNEL-positive cells decreased in rPOA/pVEGF-injected heart tissue. According to these data, although rPOA or PEI affect tissue cytotoxicity, it can be overcome through these therapeutic complexes, suggesting that cell death can minimized in a PEI- or rPOA-treated infarction model.

In this study, we optimized the gene delivery conditions to treat MI model rats using rPOA/pDNA polyplexes. Nevertheless, the number of genes transferred to cells is low, whereas high gene expression was detected *in vitro*. Furthermore, the rPOA/pDNA had higher transfection efficiency and much lower cytotoxicity than PEI complexes *in vivo*, which subsequently enhanced neovascularization and cardiac tissue repair. Collectively, these data suggest that rPOA is one of primary candidates for clinical gene therapy with its highly efficient gene delivery efficiency and low cardiac toxicity; however, the comparative advantage of rPOA over the other gene carrier systems should be continually assessed.

## Supporting Information

S1 Fig Characterization of rPOA/pVEGF polyplexes. (A) Gel retardation assay of rPOA/pVEGF polyplexes prepared at various weight ratios. (B, C) Sizes and zeta-potentials of rPOA polyplexes. (D) Size distribution as a function of intensity. Data represent the mean ± SD. rPOA, reducible poly(oligo-d-arginine).
(TIF)

S2 Fig VEGF expression levels in hypoxic and ischemic H9c2 cells and myocardium. (A) VEGF expression in cells transfected with rPOA or PEI carriers under normoxic and hypoxic conditions. VEGF expression levels were measured by ELISA. *P < 0.001, **P < 0.00001 vs. naked DNA. (B) VEGF expression in normal and ischemic myocardium injected with the rPOA/pVEGF polyplex. VEGF level was measured by ELISA one week after injection. Normal, uninjected group. Data represent the mean ± SD (n = 5 per group). *P < 0.05, **P < 0.0001.
(TIF)
