# Supplementary figures and images for: Cardiac Usage of Reducible Poly(oligo-D-arginine) As a Gene Carrier for Vascular Endothelial Growth Factor Expression
Source: PLoS One. 2015 Dec 9;10(12):e0144491. doi: 10.1371/journal.pone.0144491 (PMC4674056; doi:10.1371/journal.pone.0144491)

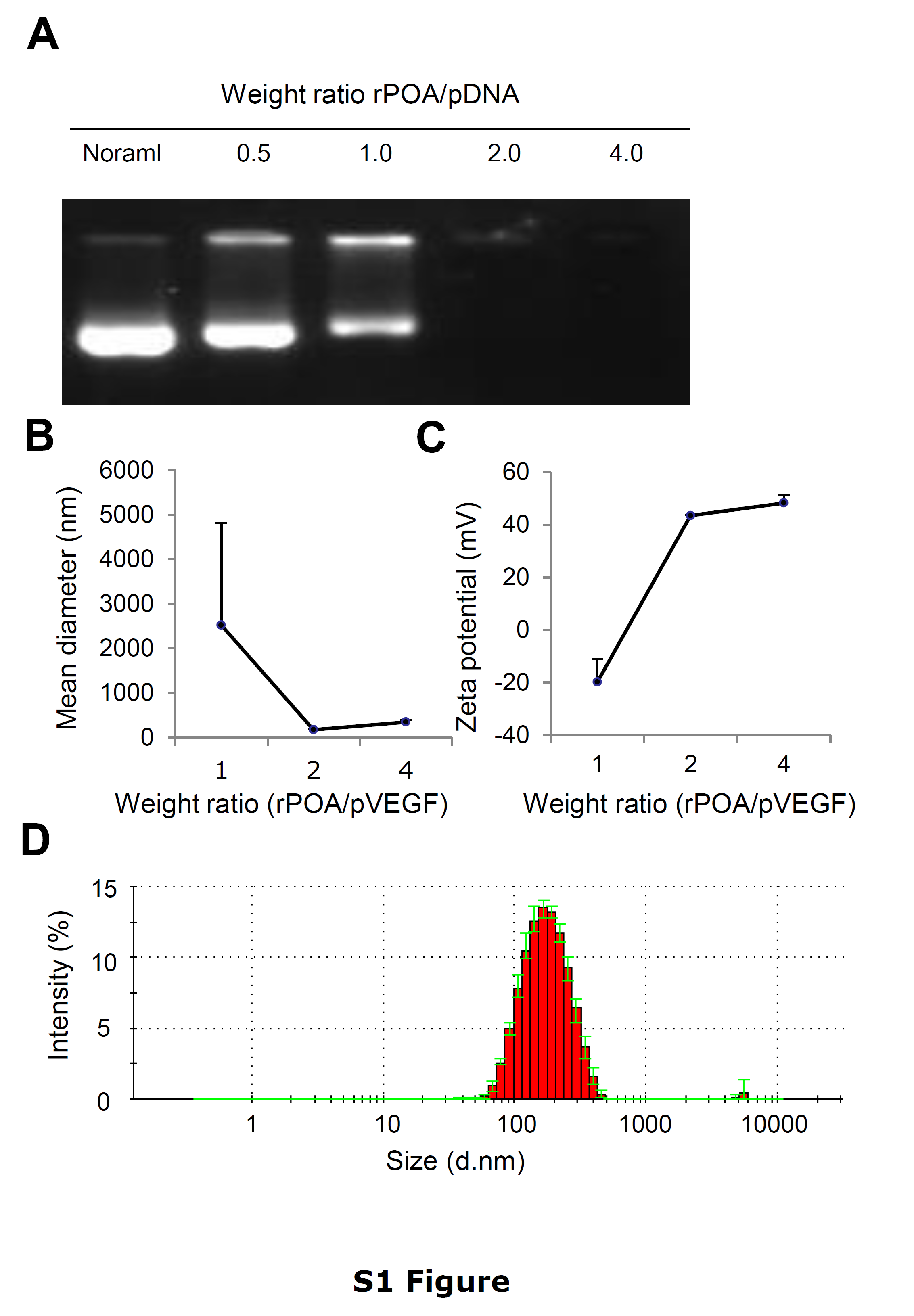

Supplement: S1 Fig — (A) Gel retardation assay of rPOA/pVEGF polyplexes prepared at various weight ratios. (B, C) Sizes and zeta-potentials of rPOA polyplexes. (D) Size distribution as a function of intensity. Data represent the mean ± SD. rPOA, reducible poly(oligo-d-arginine). (TIF) [file pone.0144491.s001.tif]

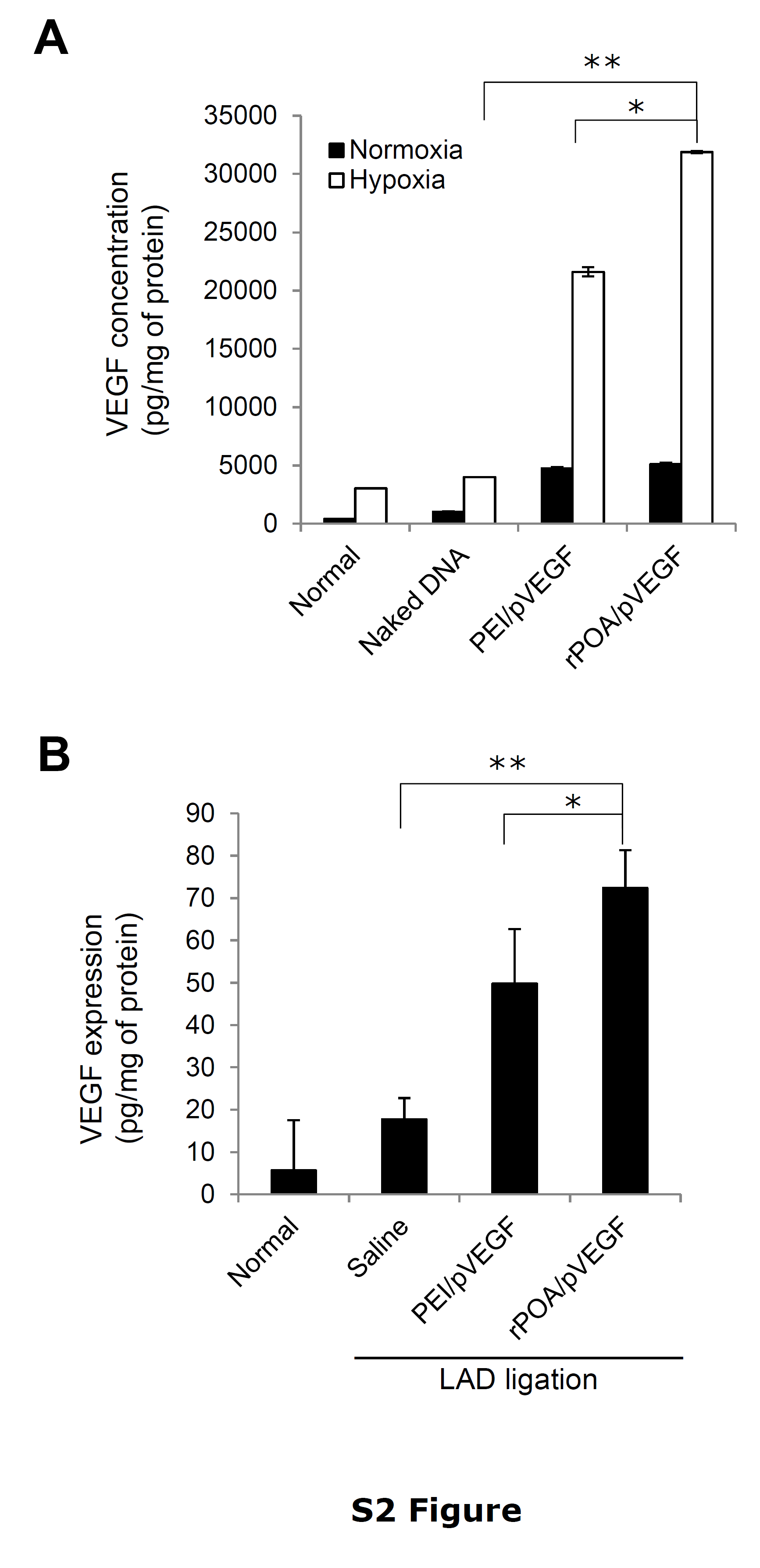

Supplement: S2 Fig — (A) VEGF expression in cells transfected with rPOA or PEI carriers under normoxic and hypoxic conditions. VEGF expression levels were measured by ELISA. *P < 0.001, **P < 0.00001 vs. naked DNA. (B) VEGF expression in normal and ischemic myocardium injected with the rPOA/pVEGF polyplex. VEGF level was measured by ELISA one week after injection. Normal, uninjected group. Data represent the mean ± SD (n = 5 per group). *P < 0.05, **P < 0.0001. (TIF) [file pone.0144491.s002.tif]
